# Lifestyle interventions in the management of systemic sclerosis: a systematic review of the literature

**DOI:** 10.1093/rap/rkae037

**Published:** 2024-03-09

**Authors:** Ioannis Parodis, Alexander Tsoi, Alvaro Gomez, Jun Weng Chow, Charlotte Girard-Guyonvarc’h, Tanja Stamm, Carina Boström

**Affiliations:** Division of Rheumatology, Department of Medicine Solna, Karolinska Institutet, Stockholm, Sweden; Department of Gastroenterology, Dermatology and Rheumatology, Karolinska University Hospital, Stockholm, Sweden; Department of Rheumatology, Faculty of Medicine and Health, Örebro University, Örebro, Sweden; Division of Rheumatology, Department of Medicine Solna, Karolinska Institutet, Stockholm, Sweden; Department of Gastroenterology, Dermatology and Rheumatology, Karolinska University Hospital, Stockholm, Sweden; Division of Rheumatology, Department of Medicine Solna, Karolinska Institutet, Stockholm, Sweden; Department of Gastroenterology, Dermatology and Rheumatology, Karolinska University Hospital, Stockholm, Sweden; Division of Rheumatology, Department of Medicine Solna, Karolinska Institutet, Stockholm, Sweden; Department of Gastroenterology, Dermatology and Rheumatology, Karolinska University Hospital, Stockholm, Sweden; Division of Rheumatology, Department of Medicine, University Hospital of Geneva and Faculty of Medicine, University of Geneva, Geneva, Switzerland; Section for Outcomes Research, Center for Medical Statistics, Informatics and Intelligent Systems, Medical University of Vienna, Vienna, Austria; Ludwig Boltzmann Institute for Arthritis and Rehabilitation, Vienna, Austria; Division of Physiotherapy, Department of Neurobiology, Care Sciences and Society, Karolinska Institutet, Stockholm, Sweden; Department of Occupational Therapy and Physiotherapy, Karolinska University Hospital, Stockholm, Sweden

**Keywords:** systemic sclerosis, lifestyle intervention, physical activity, physical exercise, patient education, self-management

## Abstract

**Objectives:**

We aimed to investigate the efficacy of lifestyle interventions for the management of SSc.

**Methods:**

We searched the MEDLINE, Embase, Web of Science and CINAHL databases in June 2021. We included studies conducted on five or more patients with SSc published between 1 January 2000 and the search date evaluating lifestyle interventions, excluding systematic reviews without meta-analyses. Critical appraisal was conducted using critical appraisal tools from the Joanna Briggs Institute. Thirty-six studies were included for full-text evaluation.

**Results:**

A total of 17 studies evaluated the effect of physical exercise alone, whereas 14 studies evaluated educational interventions for mental health management, often with physical exercise as a central component. At an aggregated level, these studies support patient education and physical exercise for the improvement of physical function, in particular hand and mouth function. Studies on diet and nutrition were few (*n* = 5) and pertained to gastrointestinal as well as anthropometric outcomes; these studies were insufficient to support any conclusions.

**Conclusion:**

Physical exercise and patient education should be considered for improving physical function in patients with SSc. These interventions can be provided alongside pharmacotherapy, but there is no evidence supporting that they can be a substitute. Further research should aim at assessing the effects of reductions of harmful exposures, including tobacco smoking and alcohol, improving sleep and enhancing social relations, three hitherto underexplored facets of lifestyle in the context of SSc.

Key messages

- The efficacy of lifestyle interventions in systemic sclerosis is underexplored.
- Patient education enhances outcomes and physical exercise improves physical function in systemic sclerosis.
- Lifestyle interventions constitute a supplement, not a substitute, to pharmacotherapy.

## Introduction

SSc is a chronic connective tissue disease that primarily affects women, commonly in their fifth decade of life, and can manifest with limited cutaneous involvement, diffuse cutaneous involvement or with no cutaneous involvement [1]. The estimated prevalence ranges from <150–443 cases per million population, being higher in regions such as southern Europe, North America and Australia [1]. Areas commonly affected by skin fibrosis are the hands and face, which often results in impaired hand function [2] and microstomia [3]. Although advances in pharmacotherapy for rheumatic diseases have been achieved during the 21st century, the guidelines for non-pharmacological management in general, and lifestyle interventions in particular, are ill-defined.

Upon examination of the literature, the definition of a lifestyle intervention itself is not clearly characterized. The American College of Lifestyle Medicine (ACLM) defines lifestyle medicine as ‘a medical specialty that uses therapeutic lifestyle interventions as a primary modality’ and lists six fundamental domains as targets of lifestyle medicine: nutrition, exercise, stress, substance abuse, sleep and relationships [4]. Analogously, the British Society of Lifestyle Medicine specifies six ‘pillars of lifestyle medicine’: healthy eating, physical activity, mental well-being, minimizing harmful substances, sleep and healthy relationships [5]. As such, a lifestyle intervention can be any intervention that covers any or all six domains, i.e. physical activity and exercise, diet and nutrition, mental health, harmful exposures, sleep and social relations.

The importance of lifestyle in the management of rheumatic diseases is gaining recognition. The EULAR recently published recommendations regarding lifestyle behaviours and work participation aimed at preventing disease progression in patients with rheumatic and musculoskeletal diseases [6]. These 18 recommendations, accompanied by five overarching principles, were derived from systematic literature reviews geared toward six ‘lifestyle exposures’, i.e. exercise, diet, weight, alcohol, smoking and work participation [6]. Recently the EULAR also issued guidelines for the non-pharmacological management of SLE and SSc [7], following a thorough systematic literature review [8]. However, this review, while comprehensive, did not distinctly isolate lifestyle interventions from other approaches. Consequently, valuable insights into lifestyle interventions targeting modifiable health factors were obscured among the multitude of non-pharmacological management strategies examined. To bridge this gap in the literature, we herein conducted a systematic literature review to address the efficacy of lifestyle interventions in different aspects of the disease course in people living with SSc.

## Methods

### Inclusion and exclusion criteria

Inclusion criteria for studies included a date of publication between 1 January 2000 and the search date, having a cohort of patients with SSc (as defined by classification criteria and/or International Classification of Diseases codes) as a population under investigation and evaluation of a lifestyle intervention. Studies were excluded if they had fewer than five participants, if they were systematic reviews without a meta-analysis, had no data on a distinct SSc patient population, were duplicates, were written in a language other than English, Spanish, or Swedish or if they did not assess an intervention that comprised one or more of the following: physical activity and exercise, diet and nutrition, mental health, harmful exposures, sleep and social relations.

### Search strategy

**Figure 1.**
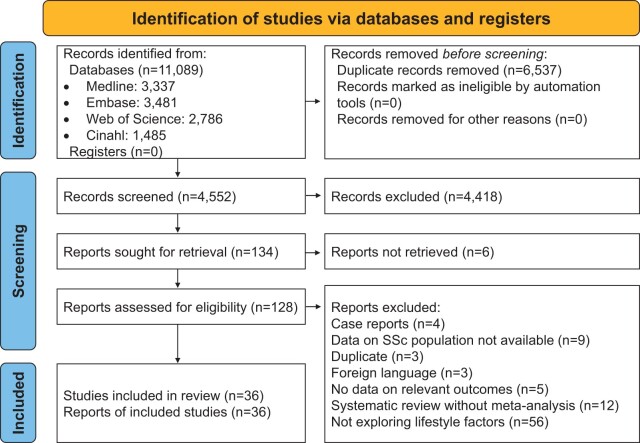
Flowchart of study selection

On 22 June 2021, the MEDLINE, Embase, Web of Science and CINAHL databases were searched for studies concerning non-pharmacological management for SSc. Two investigators (A.G. and J.C.) screened the 11 089 initial hits under supervision of one senior investigator (I.P.). Conflicts were solved upon discussion with two investigators (I.P. and C.B.). The search and study selection was documented according to the Preferred Reporting Items for Systematic Reviews and Meta-analyses statement (Fig. 1) [9].

### Data extraction

Data extraction was conducted by one researcher (J.C.) under the supervision of one senior investigator (I.P.). Data extracted included the number of participants for each study, interventions or management strategies given to both experimental and control groups, the characteristics of the comparator group, outcomes and the efficacy of the intervention. These data are provided in Supplementary Table S1, available at *Rheumatology Advances in Practice* online.

### Categorization

After data extraction and risk of bias (RoB) assessment, the studies were grouped by the category of lifestyle intervention they assessed. Studies combining exercise protocols with other educational interventions were separated from studies evaluating only physical exercise protocols, which in turn constituted their own group. Studies within the two above categories specifically evaluating the hand and mouth were further subgrouped into their own categories.

### Quality assessment and evidence grading

RoB assessment for all included articles was conducted by one researcher (A.T.) using the Joanna Briggs Institute critical appraisal (CA) tools (checklists) [10]. Since all articles were already included before quality assessment for this review, the alternatives for overall appraisal ‘include’, ‘exclude’ and ‘seek further info’ were modified to ‘robust’, ‘weak’ and ‘intermediate’, respectively. The appropriate checklist for each study was selected based on the study design. A study was deemed weak if there were six or more checklist items it did not clearly fulfil, intermediate if there were three to five checklist items it did not clearly fulfil or robust if it clearly fulfilled all checklist items but two or fewer. After CA, studies were graded by level of evidence (LoE) according to the Oxford Centre for Evidence-Based Medicine [11].

## Results

### Study characteristics

Thirty-six studies were included. Of these, 17 evaluated physical activity and exercise alone rather than in combination with other interventions [12–28]. Fourteen studies evaluated the efficacy of mental health management [29–42] and five studies assessed diet and nutrition [43–47]. Fig. 1 presents a flowchart of the study selection. Studies and their characteristics, including the number of participants, interventions, characteristics of the comparator groups and outcomes are provided in Supplementary Table S1, available at *Rheumatology Advances in Practice* online.

## Physical activity and exercise

### General

A randomized controlled trial (RCT) examining the effect of a tailored home-based exercise program (CA: intermediate; LoE: 2) found improvements in 6-min walking distance (6MWD) [48], the physical component score (PCS) of the 36-item Short Form Health Survey (SF-36) [49] and the HAQ Disability Index (HAQ-DI) [15, 50]. Two RCTs evaluating the effect of exercise on microcirculation (CA: weak; LoE: 3) found no significant impact on cutaneous vascular conductance (CVC) after 12 weeks of high-intensity interval training alone [21]; however, this produced a significant effect when combined with endurance training [22]. An RCT evaluating tai chi (CA: weak; LoE: 3) found improvements in scores relating to balance (Berg Balance Scale [51]), sleep (Pittsburgh Sleep Quality Index [52]) and fatigue (Fatigue Severity Scale [53]), but not trunk lateral endurance (trunk lateral endurance test [23, 54]. Observational studies found that lower quadriceps strength associated with worse HAQ-DI scores (CA: robust; LoE: 3) [19] and that exercise habits associated with improved scores on the HAQ-DI and the Patient-Reported Outcomes Measurement Information System [55] (PROMIS; CA: intermediate; LoE: 3) [20]. Aerobic exercise improved maximum oxygen consumption (VO_2_max) without exacerbation of skin induration, RP or digital ulcers at an 8-week follow-up (CA: intermediate; LoE: 3) [17].

### Hand

An RCT by Rannou et al. [13] (CA: robust; LoE: 2) provided 4 weeks of personalized physical therapy to the experimental group and found an improvement in the HAQ-DI and hand function measured by the Cochin Hand Function Scale (CHFS) [56] after 4 weeks compared with patients receiving usual care. However, these improvements disappeared after 12 months. Stretching programs for hands improved scores in the Canadian Occupational Performance [57] after 3 months (CA: intermediate; LoE: 2) [12] but did not improve Hand Mobility in Scleroderma (HAMIS) test [2] scores at 9 or 18 weeks, regardless of adjunct treatment with paraffin baths (CA: intermediate; LoE: 2) [14]. Two RCTs evaluating functional impairment (CA: weak; LoE: 3) showed that app-delivered occupational therapy and stretching exercises administered through a telemedicine system were efficacious in improving hand function [25] as measured by a shortened version of the Disabilities of the Arm, Shoulder and Hand questionnaire (QuickDASH) [58] and HAMIS [18]. A controlled quasi-experimental study (CA: intermediate; LoE: 3) found daily stretching exercises improved the range of motion in each finger in patients with SSc 1 month after baseline, and this improvement was maintained or further increased after 1 year from baseline [16].

### Mouth

The RCT by Rannou et al. [13] (CA: robust; LoE: 2) showed that 1 month of personalized physical therapy produced a sustained improvement in oral aperture up to 1 year from baseline. Another RCT (CA: weak; LoE: 3) found 12 weeks of an orofacial exercise protocol improved scores in the Mouth Handicap in Systemic Sclerosis index [3] up to 20 weeks from baseline [24]. An uncontrolled quasi-experimental study (CA: robust; LoE: 4) found daily mouth stretching exercises improved oral aperture 18 weeks after baseline.

## Mental health

### General

An RCT evaluating the efficacy of a self-management website (CA: weak; LoE: 3) found no differences compared with issuing an educational patient-focused book when assessing PROMIS (primary outcome) scores at 16 weeks [35]. A controlled quasi-experimental study evaluating 3 weeks of patient education through occupational therapy (CA: robust; LoE: 3) found improvements in the HAQ-DI up to 24 weeks after baseline [34].

### Hand

An RCT evaluating an educational program for self-management (CA: intermediate; LoE: 2) noted improvements in hand-related measures such as the HAMIS, Duruoz Hand Index [56], HAQ-DI and handgrip strength after 8 weeks [32]. A controlled quasi-experimental study (CA: intermediate; LoE: 3) found an educational self-management program for hands reduced the pain experienced by patients assessed using a visual analogue scale as well as improved CHFS scores after 24 weeks [36]. The protocol in this study was based on an uncontrolled study published the year before (CA: robust; LoE: 4), which also found amelioration of pain experienced by patients as well as improvements in CHFS scores after 8 weeks [42].

### Mouth

An RCT evaluating the effect of patient education with emphasis on orofacial exercises (CA: intermediate; LoE: 2) found that face-to-face training increased oral aperture more than educational material alone at 12 months after baseline in per-protocol analysis [30]. Another RCT (CA: intermediate; LoE: 2) found an increase in oral aperture after 1 month of orofacial exercise, regardless of whether they received oral hygiene advice before or after [31]. Two RCTs by Yuen et al. [29, 33] examined the effects of oral health interventions, including instruction on dental product use and orofacial exercises. One of these studies [33] found an increase in oral aperture at 3 months, but not 6 months after baseline (CA: intermediate; LoE: 2), noting low adherence to the exercise program in particular, while the other study (CA: weak; LoE: 3) assessed gingival health and found significant improvements in the Löe–Silness gingival index [59] in both groups at 6 months after baseline, but a larger improvement in the intervention group compared with controls. Yet another multifaceted oral hygiene intervention was evaluated in an uncontrolled study by Poole et al. [39] (CA: intermediate; LoE: 4) and incorporated instruction of hand exercises on top of dental hygiene and orofacial exercise instruction. After a 6-month intervention, this study noted improvements in the Patient Hygiene Performance Index (PHP) [60] after 12 months from baseline, but no improvements in upper extremity measures such as the Keitel Function Test [61] or oral aperture [39].

### Diet and nutrition

Five selected studies examined the effect of diet and nutrition (CA: four intermediate, one weak). Two RCTs on this topic examining the effects of probiotics found no significant changes in the University of California, Los Angeles Scleroderma Clinical Trial Consortium Gastrointestinal Tract Instrument (GIT-score) [62] compared with placebo after 60 days (CA: intermediate; LoE: 2) [43] and 8 weeks (CA: weak; LoE: 3) [44], respectively. However, the former study found an improvement in the GIT-score reflux component after 120 days [43] and the in latter study a decrease in Th17 cells after 8 weeks compared with placebo [44]. Conversely, one uncontrolled quasi-experimental study (CA: intermediate; LoE: 4) found that the use of probiotics associated with a significant reduction in total GIT-score as well as the reflux and bloating/distention component scores after 2 months [45].

Two quasi-experimental studies evaluated nutritional therapy. One found that nutritional support had no significant improvement in weight, body mass index (BMI) [63], energy intake or SF-36, with follow-up time points up to 12 months (CA: intermediate; LoE: 4) [46]. The other study found improvement in the abridged Patient-Generated Subjective Global Assessment [64] and a reduction in the number of patients classified as sarcopenic by DXA after 18 months (CA: intermediate; LoE: 4) [47]. The study did not find significant changes in caloric intake or macronutrient distribution in the enrolled patients [47].

## Discussion

This systematic review of the literature assessed the current evidence for lifestyle interventions as viable management strategies for people living with SSc. The main categories of intervention were physical activity and exercise, mental health and diet and nutrition. Physical exercise in general improved functional impairment and aerobic capacity, while stretching exercises of the hands and mouth efficaciously ameliorated hand impairment and microstomia. Stretching exercises of the hands and mouth were in turn often central components of educational interventions, which in principle focused on different facets of self-management. Studies on diet and nutrition showed sparse efficacy of probiotics in alleviating gastrointestinal symptoms and limited use of nutritional therapy for improving body composition. Overall, there was no rigorous investigation as to how lifestyle affects global disease activity in SSc. Furthermore, none of the included studies aimed to replace pharmacotherapy with lifestyle interventions.

These findings are largely in line with the comprehensive body of evidence compiled in the EULAR recommendations for lifestyle behaviours and work participation [6], which conclude that physical exercise can be a safe and beneficial way to improve functional impairment. However, factors such as comorbidities and disease severity warrant caution when recommending physical exercise as a part of disease management, which, as always, should be tailored to the patient. Furthermore, the findings in this review also agree that the evidence for recommending specific diets for the management of rheumatic and musculoskeletal diseases is sparse [6]. Relating to the principal components of lifestyle medicine [4, 5], there are gaps in knowledge regarding the effect of sleep, social relations and the use of harmful substances (such as nicotine, tobacco and alcohol) on SSc specifically. For these lifestyle domains, there exist EULAR recommendation sets [6, 7, 65] and other systematic reviews [8, 66].

The categorization of interventions was not absolute, as many studies employed a combination of many different intervention categories. For example, studies on nutritional therapy [46, 47] consisted of counselling and informative meetings, as in the studies on patient education. Similarly, educational interventions often had exercise programs as a central constituent [29–33, 36, 39, 42]. The complexity of stratifying studies by category implies a tendency in current research toward examining multimodal approaches when evaluating lifestyle interventions. This may be based on mechanistic reasoning that certain lifestyle interventions should produce a larger effect size when made concurrently but complicates the assessment when trying to discern the efficacy of individual interventions in isolation.

A limitation that we encountered while compiling the evidence was the lack of a structured synthesis or meta-analysis. Moreover, the overall CA was derived from assessment by only one investigator, which potentially reduces the reliability of the RoB assessment. Despite this, there are strengths to this review in the form of a generous inclusion of studies spanning a period of >2 decades with varied study designs and a conservative approach in the CA of studies, treating unclearly fulfilled criteria as unfulfilled.

Considering that most of the studies included in this review were conducted in Europe (particularly Italy) and the USA, caution should be exercised when generalizing the findings to other regions, particularly those with different healthcare systems, demographics and environmental factors. While a focus on Western countries may provide valuable insights into lifestyle interventions in SSc, extrapolating these findings to populations worldwide should be approached with caution.

In conclusion, this systematic review found physical exercise and mental health management to be efficacious lifestyle interventions for improving functional impairment in patients with SSc, which we therefore advocate should be considered for patients suffering from hand or face involvement, reduced muscle function and reduced physical fitness. Importantly, it is worth mentioning that current evidence overall supports lifestyle interventions as a complement and not a substitute to pharmacotherapy. Future studies, preferably of RCT design, are needed for exploring other aspects of lifestyle interventions, namely concerning diet and nutrition, sleep, harmful exposures and social relations, and how these potentially impact the disease course and patient experience, particularly the degree of disease activity.

## Supplementary Material

rkae037_Supplementary_Data

## Data Availability

The data underlying this article are available in the article and in its online supplementary material.
