# Supplementary material for: Lifestyle interventions in the management of systemic sclerosis: a systematic review of the literature
Source: Rheumatol Adv Pract. 2024 Mar 9;8(2):rkae037. doi: 10.1093/rap/rkae037 (PMC11001494; doi:10.1093/rap/rkae037)
Supplement: rkae037_Supplementary_Data [file rkae037_supplementary_data.pdf]

Supplementary Table S1

Study characteristics

Author, Year	Category	Participants	Intervention/management strategy[a]	Comparator	Outcome(s)[b]	SD&OA[c]	Oxford LoE[d]
**Physical activity and exercise**							
Stefanantoni, 2016 (1)	Exercise (hand)	Intervention: 15<br><br>Control: 16	P: Hand stretching exercise and weekly phone call with occupational therapist, with specific timetable for when to conduct exercise<br><br>C: Hand stretching exercise and weekly phone call with occupational therapist	Other SSc patients	COPM	11; I	2
Rannou, 2017 (2)	Exercise (multiple)	Intervention: 110<br><br>Control: 108	P: Personalized physical therapy session with physiotherapist and occupational therapist<br><br>C: Usual care	Other SSc patients	CHFS; HAQ-DI; Oral aperture	11; R	2
Gregory, 2019 (3)	Exercise (hand)	Intervention: 17<br><br>Control: 19	P: Paraffin bath and hand exercises<br><br>C: Hand exercises without wax bath	Other SSc patients	HAMIS	11; I	2
Filippetti, 2020 (4)	Exercise (general)	Intervention: 22<br><br>Control: 22	P: Home-based aerobic exercise (stationary bike), muscular endurance training (upper limbs) and stretching (hands)<br><br>C: Usual care	Other SSc patients	6MWD; HAQ-DI; SF-36	11; I	2
Mugii, 2006 (5)	Exercise (hand)	Intervention: 45<br><br>Control: 21 healthy controls	P: Self-administrated stretching<br><br>C: No intervention	Healthy controls	ROM	10; I	3
Oliveira, 2009 (6)	Exercise (general)	Intervention: 7<br><br>Control: 7	P: Aerobic exercise programme<br><br>C: Aerobic exercise programme	Healthy controls	VO_2max	10; I	3
Piga, 2014 (7)	Exercise (hand)	Intervention: 20 (10 with RA)<br><br>Control: 20 (10 with RA)	P: Stretching and mobility exercises at home using a newly developed telemedicine system<br><br>C: Home kinesiotherapy protocol	SSc patients and RA-patients	HAMIS	11; W	3
Lima, 2015 (8)	Exercise (general)	Intervention: 20<br><br>Control: 20	P: Muscle strength<br><br>C: No intervention	Healthy controls	HAQ-DI	1; R	3
Azar, 2018 (9)	Exercise (general)	Intervention: 389<br><br>Control: 363	P: Exercise habits<br><br>C: No intervention	Other SSc patients	PROMIS	5; I	3
Mitropoulos, 2018 (10)	Exercise (general)	Intervention 1: 11<br><br>Intervention 2: 11<br><br>Control: 12	P: Intervention 1: HIIT (cycling) twice a week for 12 weeks<br><br>Intervention 2: HIIT (arm cranking) twice a week for 12 weeks<br><br>C: No training protocol	Other SSc patients	CVC	11; W	3
Mitropoulos, 2019 (11)	Exercise (general)	Intervention: 16<br><br>Control: 16	P: Combined programme with HIIT and resistance training, twice weekly for 12 weeks<br><br>C: No intervention	Other SSc patients	CVC	11; W	3
Cetin, 2020 (12)	Exercise (general)	Intervention: 14<br><br>Control: 14	P: Tai Chi<br><br>C: Home exercise	Other SSc patients	Berg Balance Scale; Fatigue Severity Scale; Pittsburg	11; W	3

Author, Year	Category	Participants	Intervention/management strategy[a]	Comparator	Outcome(s)[b]	SD&OA[c]	Oxford LoE[d]
					Sleep Quality Index; Trunk Lateral Endurance Test		
Maddali Bongi, 2021 (13)	Exercise (mouth)	Intervention: 25<br><br>Control: 22	P: Home exercises for temporomandibular joint, mimic, masticatory and cervical spine muscles<br><br>C: Home exercises and combined physiotherapeutic procedures performed by a physiotherapist	Other SSc patients	MHISS	11; W	3
Murphy, 2021 (14)	Exercise (hand)	Intervention: 16<br><br>Control: 16	P: Intensive occupational therapy and app-delivered home exercise.<br><br>C: App alone	Other SSc patients	QuickDASH	11; W	3
Pizzo, 2003 (15)	Exercise (mouth)	Intervention: 10 selected among 35 patients with MMO≤30mm<br><br>Control: N/A	P: Mouth stretching exercise and oral augmentation exercise<br><br>C: N/A: intraindividual assessment	N/A; intraindividual assessment	-	10; R	4
Pinto, 2011 (16)	Exercise (general)	Intervention: 11<br><br>Control: N/A	P: Supervised, treadmill, treadmill (aerobic), stretching exercise<br><br>C: N/A: intraindividual assessment	N/A; intraindividual assessment	-	10; R	4
Defi, 2021 (17)	Exercise (general)	Intervention: 11<br><br>Control: N/A	P: High-intensity interval exercise (HIIT)<br><br>C: N/A: intraindividual assessment	N/A; intraindividual assessment	-	10; I	4
**Mental health**							
Yuen, 2012 (18)	Patient education (mouth, oral hygiene)	Intervention: 26<br><br>Control: 22	P: Orofacial exercise + multifaceted oral health intervention<br><br>C: Usual care	Other SSc patients	Oral aperture	11; I	2
Uras, 2019 (19)	Patient education (mouth)	Intervention: 31<br><br>Control: 32	P: Face-to-face training + standard information programme (i.e., brochures, DVD)<br><br>C: Educational materials alone	Other SSc patients	Oral aperture	11; I	2
Cüzdan, 2021 (20)	Patient education (mouth, oral hygiene)	Intervention: 28<br><br>Control: 28	P: Orofacial exercise programme followed by oral hygiene care advice<br><br>C: Oral hygiene care advice followed by orofacial exercise programme	Other SSc patients	Oral aperture	11; I	2
Gokcen, 2021 (21)	Patient education (hand)	Intervention: 32<br><br>Control: 30	P: Booklet, isometric hand exercise and self-administrated stretching<br><br>C: Booklet alone	Other SSc patients	DHI; HAMIS; Handgrip strength; HAQ-DI	11; I	2
Yuen, 2011 (22)	Patient education (mouth, oral hygiene)	Intervention: 26<br><br>Control: 22	P: Multi-faceted oral health intervention<br><br>C: Usual care	Other SSc patients	Löe-Silness gingival index	11; W	3
Zanatta, 2017 (23)	Patient education (general)	Intervention: 10<br><br>Control: 10	P: Informative meeting followed by occupational therapy<br><br>C: Informative meeting alone	Other SSc patients	HAQ-DI	10; R	3
Khanna, 2019 (24)	Patient education (general)	Intervention: 134<br><br>Control: 133	P: Self-management website<br><br>C: Book	Other SSc patients	PROMIS	11; W	3
Landim, 2020 (25)	Patient education (hand)	Intervention: 40<br><br>Control: 17	P: Self-management programme composed of a booklet<br><br>C: No intervention	Other SSc patients	CHFS; VAS (pain)	10; I	3

Author, Year	Category	Participants	Intervention/management strategy[a]	Comparator	Outcome(s)[b]	SD&OA[c]	Oxford LoE[d]
Samuelson, 2000 (26)	Patient education (general, self-efficacy)	Intervention: 6<br><br>Control: N/A	P: Educational programme<br><br>C: N/A: intraindividual assessment	N/A; intraindividual assessment	-	10; I	4
Brown, 2004 (27)	Patient education (general)	Intervention: 5 with SSc, 5 with SLE<br><br>Control: N/A	P: Educational programme<br><br>C: Educational programme	SLE-patients	-	9; R	4
Poole, 2010 (28)	Patient education (mouth, oral hygiene hand)	Intervention: 17<br><br>Control: N/A	P: Customized intervention for dental hygiene and upper extremity's function<br><br>C: N/A: intraindividual assessment	N/A; intraindividual assessment	KT; Oral aperture; PHP	10; I	4
Poole, 2013 (29)	Patient education (general, self-efficacy)	Intervention: 62 (49 completers, 13 non-completers)<br><br>Control: N/A	P: Mail-delivered self-management programme<br><br>C: N/A: intraindividual assessment	N/A; intraindividual assessment	-	10; I	4
Poole, 2014 (30)	Patient education (general, self-efficacy)	Intervention: 16<br><br>Control: N/A	P: Self-management website with 10 modules<br><br>C: N/A: intraindividual assessment	N/A; intraindividual assessment	-	10; R	4
Landim, 2019 (31)	Patient education (hand)	Intervention: 22<br><br>Control: N/A	P: Home based self-management programme that consisted of a booklet and information about SSc<br><br>C: N/A: intraindividual assessment	N/A; intraindividual assessment	CHFS; VAS (pain)	10; R	4
**Diet and nutrition**							
Low, 2019 (32)	Dietary therapy (probiotics)	Intervention: 19<br><br>Control: 21	P: Probiotics<br><br>C: Placebo	Other SSc patients	GIT-score	11; I	2
Marighela, 2019 (33)	Dietary therapy (probiotics)	Intervention: 37<br><br>Control: 36	P: Probiotics<br><br>C: Placebo	Other SSc patients	GIT-score; Th-17 cells	11; W	3
Frech, 2011 (34)	Dietary therapy (probiotics)	Intervention: 10<br><br>Control: N/A	P: Probiotics<br><br>C: N/A: intraindividual assessment	N/A; intraindividual assessment	GIT-score	10; I	4
Ortiz-Santamaria, 2014 (35)	Dietary therapy (nutrition)	Intervention: 9<br><br>Control: N/A	P: Individually adapted nutritional intervention<br><br>C: N/A: intraindividual assessment	N/A; intraindividual assessment	BMI; Energy intake; SF-36; Weight	10; I	4
Doerfler, 2017 (36)	Dietary therapy (nutrition)	Intervention: 18<br><br>Control: N/A	P: Medical nutrition therapy<br><br>C: N/A: intraindividual assessment	N/A; intraindividual assessment	abPGSGA; DXA	10; I	4

[a]P: Intervention/management strategy applied to population under investigation; C: Intervention/management strategy applied to participants in the comparator group. [b]Outcomes from the studies cited in the systematic review: 6MWD: Six minute walk distance; abPGSGA: abridged scored Patient-Generated Subjective Global Assessment; BMI: Body Mass Index; CHFS: Cochin Hand Function Scale; COPM: Canadian Occupational Performance Measure; CVC: Cutaneous vascular conductance; DHI: Duruoz Hand Index; DXA: Dual energy x-ray absorptiometry; FISI: Fecal Incontinence Severity Index; GIT-score: University of California, Los Angeles Scleroderma Clinical Trial Consortium Gastrointestinal Tract Instrument ; HAMIS: Hand Mobility in Scleroderma; HAQ-DI: Health Assessment Questionnaire-Disability Index; KT: Keitel Function Test; MHISS: Mouth Handicap in Systemic Sclerosis scale; PHP: Patient Hygiene Performance index; PROMIS: Patient Reported Outcomes Measurement Information System; QuickDASH: the shortened disabilities of the arm, shoulder and hand questionnaire; ROM: range of motion; SF-36: the 36-item short form health survey; STAI: State-Trait Anxiety Inventory; VAS: visual analogue scale; VO2max: maximum oxygen consumption. [c]Study design and overall appraisal (adapted from the Joanna Briggs Institute Manual for Evidence Synthesis (37)). [d]LoE: Levels of evidence according to the Oxford Centre for Evidence-Based Medicine (38).

Notation	Study design
**1**	Analytical cross-sectional study

**2**	Case-control study
**3**	Case report
**4**	Case series
**5**	Cohort study
**6**	Diagnostic test accuracy study
**7**	Economic evaluation
**8**	Prevalence study
**9**	Qualitative research
**10**	Quasi-experimental study
**11**	Randomised controlled trial
**12**	Meta-analysis, with or without systematic review

Notation	Overall appraisal
R	Robust
I	Intermediate
W	Weak

References

1. Stefanantoni K, Sciarra I, Iannace N, Vasile M, Caucci M, Sili Scavalli A, et al. Occupational therapy integrated with a self-administered stretching program on systemic sclerosis patients with hand involvement. Clin Exp Rheumatol. 2016;34 Suppl 100(5):157-61.
2. Rannou F, Boutron I, Mouthon L, Sanchez K, Tiffreau V, Hachulla E, et al. Personalized Physical Therapy Versus Usual Care for Patients With Systemic Sclerosis: A Randomized Controlled Trial. Arthritis care & research. 2017;69(7):1050-9.
3. Gregory WJ, Wilkinson J, Herrick AL. A randomised controlled trial of wax baths as an additive therapy to hand exercises in patients with systemic sclerosis. Physiotherapy. 2019;105(3):370-7.
4. Filippetti M, Cazzoletti L, Zamboni F, Ferrari P, Caimmi C, Smania N, et al. Effect of a tailored home-based exercise program in patients with systemic sclerosis: A randomized controlled trial. Scandinavian Journal of Medicine & Science in Sports. 2020;30(9):1675-84.
5. Mugii N, Hasegawa M, Matsushita T, Kondo M, Orito H, Yanaba K, et al. The efficacy of self-administered stretching for finger joint motion in Japanese patients with systemic sclerosis. Journal of Rheumatology. 2006;33(8):1586-92.
6. Oliveira NC, dos Santos Sabbag LM, de Sá Pinto AL, Borges CL, Lima FR. Aerobic exercise is safe and effective in systemic sclerosis. Int J Sports Med. 2009;30(10):728-32.
7. Piga M, Tradori I, Pani D, Barabino G, Dessi A, Raffo L, et al. Telemedicine applied to kinesiotherapy for hand dysfunction in patients with systemic sclerosis and rheumatoid arthritis: recovery of movement and telemonitoring technology. Journal of Rheumatology. 2014;41(7):1324-33.
8. Lima TR, Guimaraes FS, Carvalho MN, Sousa TL, Menezes SL, Lopes AJ. Lower limb muscle strength is associated with functional performance and quality of life in patients with systemic sclerosis. Brazilian Journal of Physical Therapy. 2015;19(2):129-36.
9. Azar M, Rice DB, Kwakkenbos L, Carrier ME, Shrier I, Bartlett SJ, et al. Exercise habits and factors associated with exercise in systemic sclerosis: a Scleroderma Patient-centered Intervention Network (SPIN) cohort study. Disability & Rehabilitation. 2018;40(17):1997-2003.
10. Mitropoulos A, Gumber A, Crank H, Akil M, Klonizakis M. The effects of upper and lower limb exercise on the microvascular reactivity in limited cutaneous systemic sclerosis patients. Arthritis Res Ther. 2018;20(1):112.
11. Mitropoulos A, Gumber A, Akil M, Klonizakis M. Exploring the microcirculatory effects of an exercise programme including aerobic and resistance training in people with limited cutaneous systemic sclerosis. Microvasc Res. 2019;125:103887.
12. Cetin SY, Calik BB, Ayan A. Investigation of the effectiveness of Tai Chi exercise program in patients with scleroderma: A randomized controlled study. Complementary Therapies in Clinical Practice. 2020;40:101181.

13. Maddali Bongi S, Passalacqua M, Landi G, Mikhaylova S, Tofani L, Del Rosso A, et al. Rehabilitation of the face and temporomandibular joint in systemic sclerosis. Therapeutic Advances in Musculoskeletal Disease. 2021;13.
14. Murphy SL, Barber M, Huang S, Sabbagh M, Cutter G, Khanna D. Intensive and App-Delivered Occupational Therapy to Improve Upper Extremity Function in Early Diffuse Cutaneous Systemic Sclerosis: A Pilot Two-Arm Trial. Rheumatology. 2021;11:11.
15. Pizzo G, Scardina GA, Messina P. Effects of a nonsurgical exercise program on the decreased mouth opening in patients with systemic scleroderma. Clinical Oral Investigations. 2003;7(3):175-8.
16. Pinto AL, Oliveira NC, Gualano B, Christmann RB, Painelli VS, Artioli GG, et al. Efficacy and safety of concurrent training in systemic sclerosis. Journal of Strength & Conditioning Research. 2011;25(5):1423-8.
17. Defi IR, Gultom C, Chorman MJ, Jennie J. High-intensity interval training can improve hand grip strength, inspiratory muscle, and quality of life in systemic sclerosis subjects. Reumatologia. 2021;59(2):98-103.
18. Yuen HK, Marlow NM, Reed SG, Mahoney S, Summerlin LM, Leite R, et al. Effect of orofacial exercises on oral aperture in adults with systemic sclerosis. Disability & Rehabilitation. 2012;34(1):84-9.
19. Uras C, Mastroeni S, Tabolli S, Masini C, Pallotta S, Teofoli P, et al. A comparison between two educational methods in the rehabilitation of the microstomia in systemic sclerosis: a randomized controlled trial. Clinical Rehabilitation. 2019;33(11):1747-56.
20. Cüzdan N, Türk İ, Çiftçi V, Arslan D, Doğan MC, Ünal İ. The effect of a home-based orofacial exercise program on oral aperture of patients with systemic sclerosis: A single-blind prospective randomized controlled trial. Archives of Rheumatology. 2021;36(2):176-84.
21. Gokcen N, Badak SO, Sarpel T, Sertdemir Y, Erken E. The Efficacy of a Home-Based, Self-Administered Hand Exercise Program for Patients With Systemic Sclerosis: A Randomized Controlled, Evaluator-Blind, Clinical Trial. JCR: Journal of Clinical Rheumatology. 2021;6:06.
22. Yuen HK, Weng Y, Bandyopadhyay D, Reed SG, Leite RS, Silver RM. Effect of a multi-faceted intervention on gingival health among adults with systemic sclerosis. Clinical & Experimental Rheumatology. 2011;29(2):S26-32.
23. Zanatta E, Rodeghiero F, Pigatto E, Galozzi P, Polito P, Favaro M, et al. Long-term improvement in activities of daily living in women with systemic sclerosis attending occupational therapy. British Journal of Occupational Therapy. 2017;80(7):417-22.
24. Khanna D, Serrano J, Berrocal VJ, Silver RM, Cuencas P, Newbill SL, et al. Randomized Controlled Trial to Evaluate an Internet-Based Self-Management Program in Systemic Sclerosis. Arthritis care & research. 2019;71(3):435-47.
25. Landim S, Bertolo M, Del Rio A, Sachetto Z, Gaino J, Poole J, et al. Sustained efficacy of a concise self-management programme for hands in systemic sclerosis: a longitudinal case-control observational study. Rheumatology. 2020;59(11):3330-9.
26. Samuelson UK, Ahlmen EM. Development and evaluation of a patient education program for persons with systemic sclerosis (scleroderma). Arthritis Care & Research. 2000;13(3):141-8.
27. Brown SJ, Somerset ME, McCabe CS, McHugh NJ. The impact of group education on participants' management of their disease in lupus and scleroderma. Musculoskeletal Care. 2004;2(4):207-17.
28. Poole J, Conte C, Brewer C, Good CC, Perella D, Rossie KM, et al. Oral hygiene in scleroderma: The effectiveness of a multi-disciplinary intervention program. Disability & Rehabilitation. 2010;32(5):379-84.
29. Poole JL, Skipper B, Mendelson C. Evaluation of a mail-delivered, print-format, self-management program for persons with systemic sclerosis. Clinical Rheumatology. 2013;32(9):1393-8.
30. Poole JL, Mendelson C, Skipper B, Khanna D. Taking charge of systemic sclerosis: a pilot study to assess the effectiveness of an internet self-management program. Arthritis care & research. 2014;66(5):778-82.
31. Landim SF, Bertolo MB, Marcatto de Abreu MF, Del Rio AP, Mazon CC, Marques-Neto JF, et al. The evaluation of a home-based program for hands in patients with systemic sclerosis. Journal of Hand Therapy. 2019;32(3):313-21.
32. Low AHL, Teng GG, Pettersson S, de Sessions PF, Ho EXP, Fan Q, et al. A double-blind randomized placebo-controlled trial of probiotics in systemic sclerosis associated gastrointestinal disease. Seminars in Arthritis & Rheumatism. 2019;49(3):411-9.
33. Marighela TF, Arismendi MI, Marvulle V, Brunialti MKC, Salomao R, Kayser C. Effect of probiotics on gastrointestinal symptoms and immune parameters in systemic sclerosis: a randomized placebo-controlled trial. Rheumatology. 2019;58(11):1985-90.
34. Frech TM, Khanna D, Maranian P, Frech EJ, Sawitzke AD, Murtaugh MA. Probiotics for the treatment of systemic sclerosis-associated gastrointestinal bloating/ distention. Clin Exp Rheumatol. 2011;29(2 Suppl 65):S22-5.

35. Ortiz-Santamaria V, Puig C, Soldevillla C, Barata A, Cuquet J, Recasens A. Nutritional support in patients with systemic sclerosis. Reumatologia Clinica. 2014;10(5):283-7.
36. Doerfler B, Allen TS, Southwood C, Brenner D, Hirano I, Sheean P. Medical Nutrition Therapy for Patients With Advanced Systemic Sclerosis (MNT PASS): A Pilot Intervention Study. Jpen: Journal of Parenteral & Enteral Nutrition. 2017;41(4):678-84.
37. (Editor) AE, (Editor) MZ. JBI Manual for Evidence Synthesis JBI2020 [Available from: https://synthesismanual.jbi.global.
38. Howick J, Chalmers I, Glasziou P, Greenhalgh T, Heneghan C, Liberati, et al. The Oxford Levels of Evidence 2 2011 [Available from: https://www.cebm.ox.ac.uk/resources/levels-of-evidence/ocebm-levels-of-evidence.
